# Women's and healthcare providers’ perceptions of long‐term complications associated with hypertension and diabetes in pregnancy: a qualitative study

**DOI:** 10.1111/1471-0528.15847

**Published:** 2019-08-16

**Authors:** S Nagraj, L Hinton, D Praveen, S Kennedy, R Norton, J Hirst

**Affiliations:** ^1^ The George Institute for Global Health University of Oxford Oxford UK; ^2^ Nuffield Department of Women's & Reproductive Health Level 3 Women's Centre John Radcliffe Hospital University of Oxford Oxford UK; ^3^ Nuffield Department of Primary Care Health Sciences Radcliffe Observatory Quarter University of Oxford Oxford UK; ^4^ The George Institute for Global Health Hyderabad India

**Keywords:** Anaemia in pregnancy, gestational diabetes, global health, hypertensive disorders of pregnancy, low resource settings, pre‐eclampsia

## Abstract

**Objectives:**

A diagnosis of hypertensive disorders during pregnancy (HDPs) or gestational diabetes mellitus (GDM) is highly predictive of women at increased risk of developing chronic hypertension, Type 2 diabetes, and cardiovascular disease. This study investigates perceptions of women and healthcare providers in rural India regarding these long‐term risks.

**Design:**

Qualitative study using modified grounded theory.

**Setting:**

Two states in rural India: Haryana and Andhra Pradesh.

**Population:**

Pregnant and postpartum women, community health workers (CHWs), primary care physicians, obstetricians, laboratory technicians, and healthcare officials.

**Methods:**

In‐depth interviews and focus group discussions explored: (1) priorities for high‐risk pregnant women; (2) detection and management of HDPs and GDM; (3) postpartum management, and (4) knowledge of long‐term sequelae of high‐risk conditions. A thematic analysis was undertaken.

**Results:**

Seven focus group discussions and 11 in‐depth interviews (*n* = 71 participants) were performed. The key priority area for high‐risk pregnant women was anaemia. Blood pressure measurement was routinely embedded in antenatal care; however, postpartum follow up and knowledge of the long‐term complications were limited. GDM was not considered a common problem, although significant variations and challenges to GDM screening were identified. Knowledge of the long‐term sequelae of GDM with regard to an increased risk of Type 2 diabetes and cardiovascular disease among doctors was minimal.

**Conclusions:**

There is a need for improved education, standardisation of testing and postpartum follow up of HDPs and GDM in rural Indian settings.

**Funding:**

SN is supported by an MRC Clinical Research Training Fellowship (MR/R017182/1). The George Institute for Global Health Global Women's Health programme provided financial support for the research assistant and fieldwork costs in India.

**Tweetable abstract:**

Improved education and postpartum care of women with hypertension and diabetes in pregnancy in rural India are needed to prevent long‐term risks.

## Tweetable abstract

Improved education and postpartum care of women with hypertension and diabetes in pregnancy in rural India are needed to prevent long‐term risks.

## Introduction

India is undergoing a major epidemiological transition towards non‐communicable diseases (NCDs).^1^ Cardiovascular disease (CVD) accounts for most deaths^2^; onset is typically early,^3^ and case fatality rates are high.^4^, ^5^ CVD is the leading cause of death in women in India.^6^ Pregnancy‐related conditions such as pre‐eclampsia and gestational diabetes mellitus (GDM) are associated with significant perinatal mortality and morbidity,^7^, ^8^ and carry independent risks for future cardiometabolic disorders in mothers.^9^, ^10^, ^11^, ^12^, ^13^ Women with pre‐eclampsia have an almost four‐fold risk of developing chronic hypertension post‐pregnancy, and approximately a two‐fold increased risk of ischaemic heart disease and stroke.^9^ Indian women are at increased risk of developing GDM; they are more likely to develop Type 2 diabetes (T2DM) at an earlier age and at a lower body mass index; and have a higher risk of coronary artery disease compared with Caucasians.^14^, ^15^, ^16^

Two‐thirds of the Indian population live in rural areas.^17^ Women in rural settings are particularly vulnerable to complications due to limited awareness of the risk factors for cardiometabolic disorders, and limited healthcare access and quality. Most antenatal care (ANC) in this context is delivered by Community Health Workers (CHWs) known as Auxiliary Nurse Midwives (ANMs).^18^ ANMs work in teams with other CHWs, known as Accredited Social Health Activists (ASHAs) and Anganwadi workers, and in partnership with their local primary health centre (PHC). ASHAs are based in the villages they serve and are often the first point of contact, providing an important role linking women to health services. Government schemes ensure free monthly ANC (including diagnostics) at a community health centre on the 9th of each month,^19^ and four postnatal visits are recommended (rising to up to seven in complicated cases).

We undertook a qualitative study in rural areas of two culturally and socio‐economically diverse states (Haryana and Andhra Pradesh). Located in northern India, Haryana has above‐average rates of gender and social inequality.^20^ Andhra Pradesh is located in South‐eastern India; 22% of its rural population are affected by multi‐dimensional poverty,^21^ and the prevalence rates of hypertension and diabetes are high.^22^ The population studied were mainly farming communities. In these two diverse rural areas, we explored (1) community priorities for high‐risk pregnant women; (2) detection and management of Hypertensive Disorders of Pregnancy (HDPs) and GDM; (3) postpartum management; and (4) knowledge of long‐term sequelae of women with high‐risk conditions.

## Methods

We undertook semi‐structured interviews and focus group discussions with key stakeholders involved in women's health during and after pregnancy, in rural parts of two diverse states in India (Haryana and Andhra Pradesh). Ethical approval was obtained from the Oxford Tropical Research & Ethics Committee (OxTREC), and The George Institute Ethics Committee, India. Patients were not involved in the development of the research.

### Participant recruitment

Key stakeholders in the health system (including government representatives, primary and secondary care doctors, CHWs, laboratory technicians, and pregnant and postpartum women) were recruited through purposive sampling and snowballing of key contacts.

District government representatives responsible for healthcare in the state were approached through The George Institute for Global Health (TGI) in Delhi and Hyderabad, for Haryana and Andhra Pradesh, respectively. Primary care physicians at two PHC facilities in each study area were approached to participate in in‐depth interviews. Obstetricians were identified through snowballing of key secondary care referral contacts known to the primary care physicians. CHWs were identified and recruited through the primary care physicians. Laboratory technicians were interviewed opportunistically during PHC visits through snowballing of contacts.

Women with both complicated and uncomplicated pregnancies, and postpartum women who had delivered a baby in the last 2 years were recruited for focus group discussions by their CHWs. All participants gave their written informed consent to participate in the study.

### Conducting interviews and focus groups

Topic guides were developed for interviews and focus group discussions. The guides were reviewed by the research team prior to use but were not piloted. All interviews and focus group discussions were audio‐recorded with consent for transcription and translation. Field notes were made during and immediately after the interviews or focus groups. Interviews and focus group discussions were conducted by a female researcher (SN), trained in qualitative methods, with the help of a research assistant from TGI. Each in‐depth interview or focus group discussion lasted 30–60 minutes, and was conducted in English when possible or in the local languages of Haryanvi (Haryana) and Telugu (Andhra Pradesh), with simultaneous translation by a research assistant.

Transcripts of the recordings were translated into English (from Haryanvi and Telugu) by a professional transcriber in India. Transcripts were validated for accuracy by a local research assistant based at TGI, India. Data were coded by the lead researcher (SN), with the assistance of an experienced qualitative researcher (LH). An inductive, modified‐grounded theory approach was adopted, using guidance for systematic, theory‐driven data analysis outlined by Corbin and Strauss.^23^ Themes were derived from the data rather than in advance. NVIVO v12 (QSR, International) software was used to facilitate data coding.

### Triangulation of results

To ensure triangulation of results, we collected data from several sources (key stakeholders) in different ways (interviews and focus groups) to explore how the results converged and complemented each other and any outlying cases.^24^ Disputes regarding the coding of themes or the findings were resolved by discussion between two researchers (SN and LH).

### Reflexivity

The lead researcher (SN) is a DPhil student with a clinical background in primary care. She entered each interview or focus group with an understanding of the principle of reflexivity,^25^ and took notes and kept a diary of the fieldwork to allow greater transparency regarding her thought processes. SN used these notes when objectively analysing the data arising from the study, comparing her initial thoughts with objective evidence and data from the interview/focus group transcripts.

## Results

A total of 71 participants (3 Government officials; 4 Obstetricians; 4 Primary Care Physicians; 33 CHWs (aged 29–54 years); 24 pregnant/postpartum women (aged 20–35); and 3 laboratory technicians) participated in seven focus group discussions and 11 in‐depth interviews. All the participants completed the study. Please see Table 1 for overview of participants and interviews/focus groups.

**Table 1:** Participants and numbers of focus group discussions and interviews

Study site	Haryana	Andhra Pradesh	Total number of participants
**Participants**	**Number of focus group discussions with (number of participants)**		
Pregnant women	1 (4)	2 (20)	24
ASHAs and AWWs	1 (8)	1 (10)	18
ANMs	1 (4)	1 (11)	15
	**Number of in‐depth Interviews with (number of participants)**		
PCPs	1 (2)	2 (2)	4
Obstetricians	1 (2)	2 (2)	4
Laboratory Technicians	1 (2)	1 (1)	3
Government officials	2 (2)	1 (1)	3
Total	7 Focus Group Discussions and 11 In‐Depth Interviews		71

ANM, Auxiliary Nurse Midwife; ASHA, Accredited Social Health Activist; AWW, Anganwadi worker; PCP, Primary Care Physician.

A summary of the main themes and subheadings arising from the qualitative analysis is given in Table 2.

**Table 2:** Themes and results

Theme	Subthemes	Quotes
Priorities of care	Anaemia	1. “The main priority in high risk pregnancy is in our area anaemia….anaemia is the highest priority.”– Interview; Government official
	Frustrations of healthcare professionals	2. “It is a constant effort… I mean, I keep on counselling,…counselling…we keep on saying every day, and about what can happen – like the side effects of continuous hypertension, continuous diabetes, [during & after pregnancy]”—Interview; Primary Care Physician
	Serious nature of HDPs	3. “Due to high BP [in pregnancy], IUD [intrauterine death] can happen.”—Focus Group; ANM
	Low prevalence of GDM	4. “It's rare that these (gestational diabetes) patients come. In spite of screening, they are not detected.”– Interview; Obstetrician
Detection & Management of HDPs and GDM	Routine BP measurement	5. “They [ANMs] have the BP apparatus, and it's a routine habit whenever an antenatal patient comes to them, whether she is coming 10 times, 12 times…”—Primary Care Physician
	Variations in screening for GDM	6. “Every woman isn't tested for blood sugar. Every woman is sent to the GH [General Hospital], but even there, blood sugar isn't tested.”—Focus Group; ASHA worker.
	Lack of awareness & knowledge of GDM guidelines.	7. “Most of them [pregnant women] are coming only after taking food so we rely on the random blood sugar sample. Mostly above 160 [mg/dl], we consider her to be referred.”– Interview; Primary Care Physician
	Challenges to rural screening, treatment & monitoring of GDM	8. “OGTT is very useful in pregnancy but we don't have that because this is a rural area and we don't have that glucose and all… and we have to do that hourly [blood test] and then 60 minutes after that…”—Interview; Primary Care Physician
	Variations in clinical practices/Lack of standardisation	9. “…And what month of pregnancy did you have the test done for sugar?” Pregnant Woman 1: “Three months pregnancy.” Pregnant Woman 2: “For me it was 5 months.” Pregnant Woman 3: “3rd month and then 7th month”. – Focus group; Pregnant women
Postpartum management	Postpartum follow up of high‐risk women	10. “Post‐delivery we don't check their BP. If someone has any problem, then we'll check their BP.”—Focus Group; ANM
	Responsibility for postpartum care	11. “They might have some problems [after delivery], the ASHA worker does six visits, we usually do one visit, but for high risk cases, we do more visits.” ‐ Focus Group; ANM
Long‐term sequelae of high‐risk pregnancy conditions	Knowledge & empowerment of women	12. “Basically education is the most important part. Second part is our eating habits. We have to eat healthy, and we have to educate our kids to eat healthy.”– Interview; Government official
	Workforce constraints	13. “We have to take care of the 12 villages and 38,000 population and there are so many programmes … we are always on our toes, at meetings and all… clinically, we are getting less of time to see patients…”—Interview; Primary Care Physician

### Community priorities for high‐risk pregnant women

#### Anaemia: highest priority clinical condition in rural areas

Stakeholders prioritised anaemia in pregnancy, followed by HDPs, as the most important priorities for high‐risk pregnant women in their areas. These conditions were chosen for differing reasons: anaemia because of its high prevalence and hypertension because of the serious impacts on maternal and neonatal mortality and morbidity.

Anaemia was the most common condition affecting pregnant women at both study sites (Table 2, Quote 1). Moderate to severe anaemia was deemed the highest priority by all levels of healthcare workers and government officials. Although pregnant women recognised the potentially negative consequences of anaemia in pregnancy on their babies, they did not have the same sense of urgency or recognise the serious impacts of the condition on their own health, identified by other levels of stakeholder.

Community health workers and doctors were well informed about the causes of anaemia in pregnancy, including dietary factors, household poverty, multiple pregnancy, and helminth infection. They counselled women accordingly, regarding lifestyle changes and adherence to oral iron. Healthcare workers expressed their frustrations with regard to preventing anaemia in pregnant women.

“The patient gets pregnant multiple times, the pregnancy‐induced hypertension, gestational diabetes will increase in each and every pregnancy…Anaemia will increase….And sometimes the patients are such stubborn patients that even ASHA workers say – please come to the hospital and I will go with you to be treated – No… The patient will not come…She will come with 3 g Hb [haemoglobin of 3 g/dL], when she is going to die….then she will come.”

Interview: Obstetrician.

Pregnant women, however, reported facing several difficulties in following the advice provided by their healthcare workers, including long‐established cultural and household dietary practices, and intolerable side effects of oral iron.

#### Hypertensive disorders of pregnancy are a priority condition

Hypertension was regarded as a priority because of the potentially serious consequences to the mother and baby. Knowledge of HDPs, including pre‐eclampsia, was embedded in ANC, and the training and education of all healthcare workers. Pregnant women also understood the importance of blood pressure (BP) measurement as part of routine care. Healthcare workers demonstrated knowledge of the diagnostic criteria, symptoms, and complications of pre‐eclampsia.

“Even the baby might suffer if mother has high BP…the sac may dry up.”…“Due to high BP, IUD [Intrauterine death] can happen.

” Focus group: ANMs

During the focus groups, CHWs discussed cases of women who had reported normal BP throughout pregnancy, and presented in labour with a high BP, with serious consequences, including stillbirth (Table 2, Quote 3). In spite of this, there was a paradoxical misconception that high peripartum BP could also be associated with the ‘stress’ of giving birth, that is, not a pathology that required medical attention and follow up from either specialists or CHWs.

“…at the time of delivery, there will be increase in BP in most of them [the women] and that too due to apprehension, stress, and due to the labour pains and all of the women will have that…”

Interview: Obstetrician

#### Gestational diabetes is an area of low concern in rural areas

Although recognised as a high‐risk condition, GDM was considered low priority at both study sites, with very few cases reported by women, healthcare workers, and government officials (Table 2, Quote 4).

### Detection and management of HDPs and GDM

Blood pressure measurement was embedded in routine ANC, and the experiences of healthcare workers and pregnant women alike (Table 2, Quote 5). ASHAs saw it as part of their role to counsel women about their BP and its management, but not to take BP readings (although they expressed a desire to learn this skill).

Obstetricians reportedly provided advice on when to stop antihypertensives. One woman described taking medication for a short period until her BP normalised, which later had to be restarted. ASHAs conducted most home visits but home‐based BP monitoring was not conducted. The lack of home BP measurement was explained by women and healthcare workers because “ASHAs don't take BP”.

There were significant variations in testing for GDM and the diagnostic criteria used (Table 2, Quotes 6, 7, 9). The most frequent screening method reported was a random, non‐fasting, capillary blood glucose measurement performed by a laboratory technician at the PHC. If abnormal, the woman would be referred to an obstetrician. The gold standard oral glucose tolerance test (OGTT) was not commonly performed at the primary care level and was limited to secondary care contexts (Table 2, Quote 8). Limitations of performing OGTTs in rural settings included the lack of available tests and time for pregnant women and healthcare workers, given their work commitments.

“We can do [the OGTT]…but it will take time…Those two hours of waiting, taking a sample again… and all that… I've seen a patient tested there… it takes two hours for each patient… waiting for hours is difficult in villages… There will be people who go to work.”

Focus group: ANM

Alternatives to the OGTT were offered: pregnant women were asked to attend the PHC with a roti (Indian bread), and capillary or venous blood samples were taken before and after eating it. These practices did not follow any established guidelines, but had been locally devised.

Pregnant women described having their blood sugar measured; however, there was a lack of understanding about why, how, and when the test needed to be performed in pregnancy. In contrast with how BP measurement during ANC had become routine practice, there were not similar expectations for GDM screening.

The management of GDM varied between study sites. In Andhra Pradesh, insulin was first‐line treatment, although this was difficult to implement in the villages because refrigeration and home blood glucose monitoring were not available. In one particular village, with a more affluent population, the laboratory technician made reference to everyone having a refrigerator and using glucometers at home. This finding was not replicated in other villages.

“There is a lot of awareness here… this is a rich village… there is no one who doesn't have a fridge…. Already older patients have glucometers with them… most of them have relatives staying in America.”

Interview: Laboratory technician

In contrast, in Haryana, the first line management of GDM was metformin, which was free of charge through government schemes, and in this setting, did not require blood glucose monitoring.

“Metformin is free of cost here for patients…. the patients that come here are poor, first they want to come here as the services are free.”

Interview: Obstetrician

When asked about the new Government of India 2018 GDM guidelines (which suggest CHWs perform OGTTs and capillary blood glucose measurements in the villages),^26^ primary care physicians and obstetricians felt community‐based screening for GDM would be valuable; however, they did not trust the ASHAs to perform these tests. Doctors saw the ASHA's main role to be motivating the women to come to a health facility, rather than delivery of clinical skills at home.

### Postpartum management of high‐risk women

Following delivery, it was cultural practice for women at both study sites to remain at home for the first 40 days. Their main point of contact during this period was ASHAs. Women would not leave their house unless the baby had a problem requiring hospital care or they were advised to do so by the ASHA.

Although obstetricians counselled women about the chance of recurrence of HDPs and GDM in subsequent pregnancies, and recommended postpartum checking of BP and blood glucose in high‐risk women, they acknowledged that postpartum blood glucose measurements were rarely performed. However, they presumed BP was checked postpartum, although the women with experience of HDPs reported their BP was not measured after returning home (Table 2, Quote 10).

Obstetricians felt that postpartum care was the responsibility of the ANMs. However, in reality, ASHAs were providing the majority of postpartum care in the villages. Pregnant women expressed a great deal of trust in their ASHAs and appreciated the continuity of care provided.

“Almost every day ASHA used to come home… she used to check baby's temperature and all….My house is on the way to ASHA to her workplace.”… “We trust ASHA. We get a lot of benefits from ASHA.”

Focus group: postpartum women

### Knowledge of long‐term sequelae of women with high‐risk conditions

#### Knowledge and empowerment

Pregnant women felt unsure about the long‐term consequences of high‐risk conditions in pregnancy, in terms of both the impact on future pregnancies and their own future health. They expressed a desire for more education in these areas, and an eagerness to learn and empower themselves. They felt the people best placed to educate them were doctors, as they felt doctors had the most knowledge.

Although doctors recognised links between these high‐risk conditions and future pregnancies, when specifically asked, primary care physicians and obstetricians were unaware of the associations between HDPs and GDM, and future cardiometabolic disorders. In contrast, CHWs, who live in the villages with the women, understood the link.

“There are chances she might have problems later in her life… like heart attack, brain stroke. BP keeps on coming on and off.”…. “We can advise yoga, regular usage of medicines, proper diet.”

Focus group: ASHA

Community health workers reported that it was a common finding that women over the age of 30 in their villages were developing hypertension, diabetes, and heart disease.

“Nowadays, blood pressure and diabetes have become so common that we check their BP and sugar compulsorily [in adulthood].

” Focus group: ANM

#### Workforce limitations

Both government officials and obstetricians reported the overcrowding of secondary and tertiary delivery centres. They highlighted severe workforce shortages affecting delivery of maternity care in rural areas, with advertised positions still unfilled. They acknowledged these shortages limited the time they could spend educating and empowering pregnant women, and delivering postnatal care—a task shared with CHWs (Table 2, Quote 13).

## Discussion

### Main findings

To our knowledge, this is the first qualitative study exploring the views and knowledge of women and key healthcare stakeholders regarding the long‐term cardiometabolic sequelae of high‐risk conditions in pregnancy in rural India.

Our findings suggest that anaemia is the highest priority condition for high‐risk pregnant women at both study sites. Our study also highlights that knowledge of HDPs and BP measurement has become embedded in routine ANC practice. In contrast, screening practices for GDM are highly inconsistent and are rarely evidence‐based. The lack of uniformity in diagnostic criteria for GDM was mirrored by the lack of routine GDM testing in ANC. Postpartum follow up and testing of women with HDPs and GDM were not routinely performed.

There was limited awareness of the long‐term cardiometabolic sequelae of HDPs and GDM and the need to follow up high‐risk women at either primary or secondary care level. However, CHWs (by virtue of the continuity of care they provide) had witnessed the long‐term sequelae of both HDPs and GDM in their villages.

### Strengths and limitations

This study was rigorous in its methodology and adopted a theory‐driven approach to data analysis. A representative sample of all stakeholders ensured a wide range of data sources from which to draw conclusions. Although data saturation was reached at these two sites, the views expressed may not be representative of these communities as a whole. Whilst most of the in‐depth interviews were conducted individually as per study protocol, at one site, primary care physicians/obstetricians/laboratory technicians were interviewed in pairs at their request.

### Interpretation in light of evidence

Anaemia was identified as the most common and important high‐risk condition affecting pregnant women at both study sites. This finding is supported by several studies,^27^, ^28^, ^29^, ^30^ which have highlighted that >50% of pregnant women in India are anaemic.^31^ The Government of India has recognised the importance of the early recognition and treatment of anaemia throughout a woman's life‐course and have recently launched the ‘Anemia Mukt Bharat’ (Anaemia Free India) campaign.^32^ This includes comprehensive guidelines for the diagnosis and management of anaemia in pregnancy, including free medication (iron folate tablets and intravenous iron sucrose), and resources to improve diet and adherence to medication.^32^ Our study highlighted a mismatch between behaviour change interventions provided by healthcare workers around anaemia prevention and the constraints of pregnant women within their socio‐cultural environments. This finding reinforces the results of Chatterjee and Fernandes,^33^ and highlights the importance of adopting wider societal and family‐based awareness programmes for anaemia in pregnancy, particularly targeting key household members, in addition to individually focused opportunistic counselling.

Gestational diabetes mellitus has been the key priority area for funding and research within the Indian context.^34^, ^35^, ^36^ Most studies of GDM in India have focused on the growing semi‐urban and urban populations, where there are significant differences to rural settings, in terms of physical characteristics (body mass index), the nature of daily labour, and socio‐economic status.^37^ Although the finding of a low reporting of GDM in our study might be, in part, explained by diagnostic inconsistencies, it is unlikely to account for the whole picture. Under‐nutrition leading to maternal anaemia, rather than over‐nutrition and obesity (associated with Type 2 diabetes), was considered the highest priority for pregnant women in our study. The Government of India has recently launched new guidelines for GDM testing in rural settings, recommending community‐based OGTTs.^26^ However, the extent to which these guidelines can be operationalised within our study settings is limited. Alternative guidelines for GDM testing in rural areas exist but are based on small‐scale research studies, relying on the presence of funding.^38^ The barriers to the widespread implementation of GDM guidelines and successful integration into the existing healthcare system in rural areas, are significant. Strengthening of health systems, through supporting rural PHCs, provision of adequate training, education, equipment, supply chains of medications and diagnostics, is required before high‐quality implementation of these guidelines is possible.

Postpartum management and long‐term follow up of high‐risk pregnant women were found to be lacking in our study. As socio‐cultural practices around childbirth in rural India often require the woman to stay at home for the first 6 weeks postpartum, CHWs are ideally placed to deliver community‐based interventions to engage and follow up women in their homes, and ensure continuity of care. Home‐based BP monitoring by CHWs has been shown to be feasible and effective in several studies, both within and outside the context of pregnancy.^39^, ^40^, ^41^, ^42^, ^43^ With the correct support and supervision, task‐sharing postpartum care with CHWs may help address the significant workforce shortages affecting specialist obstetricians in rural India.

## Conclusions

Given the high rates of hypertension, CVD, and conversion of GDM to T2DM for women in India, there is a need for improved education of health providers, as well as standardisation of testing and diagnosis of HDPs and GDM in rural Indian settings. Further work is particularly needed to raise awareness among rural women and healthcare workers at all levels of the postpartum period and long‐term health of women with a history of hypertension and GDM. Early identification and counselling of women at high risk of cardiometabolic disorders through innovative community‐level interventions may offer opportunities to address the escalating burden of these conditions.

### Disclosure of interests

There are no competing interests declared. Completed disclosure of interests forms are available to view online as supporting information.

### Contribution to authorship

SN was responsible for the conception, planning, delivery, analysis, and writing up of the paper. LH contributed to the planning of focus groups/interviews, qualitative analysis and provided feedback on the draft paper. DP contributed to the planning and delivery of the study, and provided feedback on the draft paper. JH contributed to the planning of the study and provided feedback on the study design, results, and drafting of the paper. RN and SK helped revise the paper.

### Details of ethics approval

Ethical approval for the study was obtained from the Oxford Tropical Research Ethics Committee (OxTREC), UK (date of approval 01/02/2018; Reference no: 506‐18); and The George Institute Ethics Committee, India (date of approval 07/03/2018; Reference no: 004/2018).

### Funding

SN is supported by a Medical Research Council Clinical Research Training Fellowship (MR/R017182/1). The George Institute for Global Health Global Women's Health programme provided financial support for the research assistant and fieldwork costs in India.
